# Supplementary material for: Modeling glioblastoma heterogeneity as a dynamic network of cell states
Source: Mol Syst Biol. 2021 Sep 16;17(9):e10105. doi: 10.15252/msb.202010105 (PMC8444284; doi:10.15252/msb.202010105)
Supplement: Supplementary file 6 — Source Data for Figure 5 [file MSB-17-e10105-s004.zip › Figure5A_sourcedata/GSEA_3071/nosigdb_stateIV.GseaPreranked.1622795164626/FELDMAN_2020_G1.html]

Details for gene set FELDMAN\_2020\_G1[GSEA]

|  || Dataset | state53071 |
| Phenotype | NoPhenotypeAvailable |
| Upregulated in class | na\_neg |
| GeneSet | FELDMAN\_2020\_G1 |
| Enrichment Score (ES) | -0.62055564 |
| Normalized Enrichment Score (NES) | -3.389021 |
| Nominal p-value | 0.0 |
| FDR q-value | 0.0 |
| FWER p-Value | 0.0 |
Table: GSEA Results Summary

  

Fig 1: Enrichment plot: FELDMAN\_2020\_G1      
 Profile of the Running ES Score & Positions of GeneSet Members on the Rank Ordered List

  

| PROBE | GENE SYMBOL | GENE\_TITLE | RANK IN GENE LIST | RANK METRIC SCORE | RUNNING ES | CORE ENRICHMENT || 1 | S100B |  |  | 12 | 2.461 | 0.0185 | No |
| 2 | PTN |  |  | 25 | 2.140 | 0.0342 | No |
| 3 | SCRG1 |  |  | 28 | 2.056 | 0.0512 | No |
| 4 | IGFBP3 |  |  | 59 | 1.794 | 0.0605 | No |
| 5 | PRCP |  |  | 242 | 1.169 | 0.0351 | No |
| 6 | SCG2 |  |  | 294 | 1.040 | 0.0340 | No |
| 7 | ARL6IP1 |  |  | 458 | 0.849 | 0.0096 | No |
| 8 | DLG1 |  |  | 520 | 0.798 | 0.0045 | No |
| 9 | MALAT1 |  |  | 739 | 0.638 | -0.0323 | No |
| 10 | PTTG1 |  |  | 2470 | -0.266 | -0.3652 | No |
| 11 | LMNB1 |  |  | 2487 | -0.268 | -0.3661 | No |
| 12 | RNASEH2A |  |  | 2595 | -0.282 | -0.3844 | No |
| 13 | CMC2 |  |  | 2632 | -0.287 | -0.3890 | No |
| 14 | HMGB1 |  |  | 2819 | -0.308 | -0.4224 | No |
| 15 | SMC4 |  |  | 3035 | -0.337 | -0.4612 | No |
| 16 | ILF2 |  |  | 3347 | -0.381 | -0.5182 | No |
| 17 | TUBA1B |  |  | 3357 | -0.383 | -0.5168 | No |
| 18 | TROAP |  |  | 3621 | -0.432 | -0.5641 | No |
| 19 | CKAP2 |  |  | 3633 | -0.433 | -0.5625 | No |
| 20 | KNSTRN |  |  | 3646 | -0.435 | -0.5612 | No |
| 21 | TMEM106C |  |  | 3669 | -0.438 | -0.5617 | No |
| 22 | KIF22 |  |  | 3832 | -0.472 | -0.5891 | No |
| 23 | CMTM7 |  |  | 3869 | -0.480 | -0.5921 | No |
| 24 | CALM2 |  |  | 3909 | -0.489 | -0.5955 | No |
| 25 | PTGES3 |  |  | 3953 | -0.498 | -0.5996 | No |
| 26 | CENPF |  |  | 4003 | -0.511 | -0.6048 | No |
| 27 | HIST1H1E |  |  | 4011 | -0.515 | -0.6018 | No |
| 28 | HSP90AA1 |  |  | 4024 | -0.519 | -0.5997 | No |
| 29 | PHF19 |  |  | 4041 | -0.523 | -0.5984 | No |
| 30 | CYCS |  |  | 4074 | -0.532 | -0.6001 | No |
| 31 | CCNB2 |  |  | 4103 | -0.542 | -0.6010 | No |
| 32 | PLK1 |  |  | 4205 | -0.569 | -0.6158 | Yes |
| 33 | MIS18A |  |  | 4230 | -0.575 | -0.6155 | Yes |
| 34 | ANP32B |  |  | 4235 | -0.576 | -0.6115 | Yes |
| 35 | HMGB2 |  |  | 4243 | -0.578 | -0.6079 | Yes |
| 36 | BUB3 |  |  | 4249 | -0.580 | -0.6040 | Yes |
| 37 | SNRPD1 |  |  | 4254 | -0.582 | -0.5999 | Yes |
| 38 | SIVA1 |  |  | 4263 | -0.586 | -0.5965 | Yes |
| 39 | IGFBP5 |  |  | 4280 | -0.592 | -0.5946 | Yes |
| 40 | EXOSC8 |  |  | 4293 | -0.596 | -0.5919 | Yes |
| 41 | KPNA2 |  |  | 4361 | -0.621 | -0.5996 | Yes |
| 42 | DNMT1 |  |  | 4388 | -0.632 | -0.5993 | Yes |
| 43 | DDX39A |  |  | 4467 | -0.661 | -0.6088 | Yes |
| 44 | RPA3 |  |  | 4482 | -0.670 | -0.6059 | Yes |
| 45 | H2AFX |  |  | 4495 | -0.677 | -0.6025 | Yes |
| 46 | CYR61 |  |  | 4498 | -0.678 | -0.5972 | Yes |
| 47 | DHFR |  |  | 4517 | -0.688 | -0.5948 | Yes |
| 48 | DTYMK |  |  | 4566 | -0.715 | -0.5981 | Yes |
| 49 | NCAPG |  |  | 4568 | -0.716 | -0.5923 | Yes |
| 50 | DBF4 |  |  | 4570 | -0.717 | -0.5864 | Yes |
| 51 | TUBB4B |  |  | 4575 | -0.722 | -0.5811 | Yes |
| 52 | PBK |  |  | 4593 | -0.730 | -0.5782 | Yes |
| 53 | CKS2 |  |  | 4600 | -0.733 | -0.5732 | Yes |
| 54 | BIRC5 |  |  | 4618 | -0.745 | -0.5702 | Yes |
| 55 | PTMA |  |  | 4620 | -0.746 | -0.5641 | Yes |
| 56 | MIAT |  |  | 4622 | -0.748 | -0.5580 | Yes |
| 57 | DUT |  |  | 4642 | -0.762 | -0.5552 | Yes |
| 58 | USP1 |  |  | 4649 | -0.763 | -0.5499 | Yes |
| 59 | NUSAP1 |  |  | 4654 | -0.765 | -0.5443 | Yes |
| 60 | CENPH |  |  | 4679 | -0.786 | -0.5423 | Yes |
| 61 | CENPA |  |  | 4683 | -0.788 | -0.5362 | Yes |
| 62 | MCM7 |  |  | 4704 | -0.800 | -0.5333 | Yes |
| 63 | GTSE1 |  |  | 4722 | -0.808 | -0.5298 | Yes |
| 64 | KIF4A |  |  | 4727 | -0.812 | -0.5237 | Yes |
| 65 | CDCA2 |  |  | 4732 | -0.816 | -0.5176 | Yes |
| 66 | RANBP1 |  |  | 4741 | -0.823 | -0.5122 | Yes |
| 67 | RAN |  |  | 4743 | -0.824 | -0.5054 | Yes |
| 68 | KIF20B |  |  | 4745 | -0.825 | -0.4987 | Yes |
| 69 | CENPE |  |  | 4757 | -0.832 | -0.4938 | Yes |
| 70 | PDLIM7 |  |  | 4768 | -0.838 | -0.4886 | Yes |
| 71 | CKAP2L |  |  | 4769 | -0.839 | -0.4816 | Yes |
| 72 | VRK1 |  |  | 4773 | -0.842 | -0.4750 | Yes |
| 73 | NUF2 |  |  | 4782 | -0.847 | -0.4694 | Yes |
| 74 | ASPM |  |  | 4789 | -0.851 | -0.4634 | Yes |
| 75 | TMPO |  |  | 4790 | -0.851 | -0.4562 | Yes |
| 76 | CENPN |  |  | 4796 | -0.858 | -0.4500 | Yes |
| 77 | KIF23 |  |  | 4825 | -0.891 | -0.4479 | Yes |
| 78 | MKI67 |  |  | 4836 | -0.902 | -0.4422 | Yes |
| 79 | PRC1 |  |  | 4846 | -0.912 | -0.4362 | Yes |
| 80 | SMC2 |  |  | 4847 | -0.912 | -0.4285 | Yes |
| 81 | CTGF |  |  | 4852 | -0.915 | -0.4216 | Yes |
| 82 | DLGAP5 |  |  | 4857 | -0.922 | -0.4146 | Yes |
| 83 | ORC6 |  |  | 4865 | -0.931 | -0.4081 | Yes |
| 84 | GMNN |  |  | 4873 | -0.939 | -0.4015 | Yes |
| 85 | CDCA3 |  |  | 4874 | -0.940 | -0.3936 | Yes |
| 86 | HIST1H1C |  |  | 4877 | -0.940 | -0.3860 | Yes |
| 87 | CENPM |  |  | 4881 | -0.947 | -0.3786 | Yes |
| 88 | GINS2 |  |  | 4897 | -0.962 | -0.3734 | Yes |
| 89 | AURKA |  |  | 4906 | -0.973 | -0.3667 | Yes |
| 90 | FBXO5 |  |  | 4910 | -0.978 | -0.3590 | Yes |
| 91 | DNAJC9 |  |  | 4916 | -0.982 | -0.3517 | Yes |
| 92 | H2AFZ |  |  | 4919 | -0.983 | -0.3438 | Yes |
| 93 | SNRPB |  |  | 4931 | -0.996 | -0.3375 | Yes |
| 94 | CCNB1 |  |  | 4962 | -1.032 | -0.3346 | Yes |
| 95 | CEP55 |  |  | 4972 | -1.047 | -0.3275 | Yes |
| 96 | CENPW |  |  | 4978 | -1.056 | -0.3196 | Yes |
| 97 | ATAD2 |  |  | 4980 | -1.058 | -0.3109 | Yes |
| 98 | TPST2 |  |  | 4989 | -1.073 | -0.3034 | Yes |
| 99 | RRM1 |  |  | 4990 | -1.074 | -0.2943 | Yes |
| 100 | UBE2T |  |  | 5000 | -1.091 | -0.2868 | Yes |
| 101 | MAD2L1 |  |  | 5007 | -1.105 | -0.2787 | Yes |
| 102 | SPC25 |  |  | 5012 | -1.112 | -0.2700 | Yes |
| 103 | FEN1 |  |  | 5014 | -1.113 | -0.2608 | Yes |
| 104 | AURKB |  |  | 5016 | -1.118 | -0.2516 | Yes |
| 105 | TK1 |  |  | 5020 | -1.126 | -0.2427 | Yes |
| 106 | TOP2A |  |  | 5029 | -1.152 | -0.2345 | Yes |
| 107 | TPX2 |  |  | 5049 | -1.184 | -0.2282 | Yes |
| 108 | CCNA2 |  |  | 5051 | -1.186 | -0.2184 | Yes |
| 109 | DEPDC1 |  |  | 5053 | -1.193 | -0.2085 | Yes |
| 110 | NDC80 |  |  | 5055 | -1.200 | -0.1985 | Yes |
| 111 | PCNA |  |  | 5060 | -1.214 | -0.1891 | Yes |
| 112 | ESCO2 |  |  | 5067 | -1.222 | -0.1799 | Yes |
| 113 | CDC20 |  |  | 5082 | -1.249 | -0.1721 | Yes |
| 114 | CENPK |  |  | 5091 | -1.267 | -0.1629 | Yes |
| 115 | UBE2C |  |  | 5106 | -1.314 | -0.1546 | Yes |
| 116 | CCDC34 |  |  | 5111 | -1.316 | -0.1442 | Yes |
| 117 | ZWINT |  |  | 5115 | -1.321 | -0.1337 | Yes |
| 118 | CKS1B |  |  | 5121 | -1.338 | -0.1233 | Yes |
| 119 | C12orf75 |  |  | 5128 | -1.356 | -0.1130 | Yes |
| 120 | TYMS |  |  | 5156 | -1.477 | -0.1058 | Yes |
| 121 | CENPU |  |  | 5159 | -1.486 | -0.0937 | Yes |
| 122 | CDK1 |  |  | 5164 | -1.512 | -0.0817 | Yes |
| 123 | ANLN |  |  | 5183 | -1.577 | -0.0718 | Yes |
| 124 | GJB2 |  |  | 5199 | -1.658 | -0.0608 | Yes |
| 125 | CLSPN |  |  | 5202 | -1.663 | -0.0471 | Yes |
| 126 | AHNAK2 |  |  | 5216 | -1.741 | -0.0349 | Yes |
| 127 | HIST1H1B |  |  | 5223 | -1.811 | -0.0208 | Yes |
| 128 | RRM2 |  |  | 5232 | -1.875 | -0.0065 | Yes |
| 129 | HIST1H4C |  |  | 5249 | -2.081 | 0.0079 | Yes |
Table: GSEA details [plain text format]

  

Fig 2: FELDMAN\_2020\_G1: Random ES distribution      
 Gene set null distribution of ES for **FELDMAN\_2020\_G1**
